# Supplementary material for: Decoding TRIP13’s Role in Gastric Cancer: Implications for Prognosis and Immune Response
Source: Biomedicines. 2025 Sep 15;13(9):2268. doi: 10.3390/biomedicines13092268 (PMC12467871; doi:10.3390/biomedicines13092268)
Supplement: Supplementary file 1 [file biomedicines-13-02268-s001.zip › biomedicines-3840849-supplementary.pdf]

Table S1. The ferroptosis-related genes.

Gene Symbol	Expression in GC	Function Related to Ferroptosis
CDKN1A	Downregulated	Regulates cell cycle and ferroptosis sensitivity
CISD1	No difference	Regulates iron uptake
EMC2	No difference	Regulates transferrin receptor
FANCD2	Upregulated	Regulates lipid peroxidation and iron metabolism
FDFT1	No difference	Regulates lipid peroxidation and iron metabolism
GPX4	Upregulated	Involves in the metabolism of lipids and antioxidants
HSPA5	Upregulated	Regulates GPX4 pathway
HSPB1	No difference	Regulates iron uptake and ROS
MT1G	Downregulated	Regulates GPX4 pathway
NFE2L2	No difference	Regulates antioxidant and iron metabolism proteins.
SAT1	No difference	Regulates lipid peroxidation and iron metabolism
SLC1A5	Upregulated	Regulates antioxidants and ferroptosis
SLC7A11	No difference	A key regulator of intracellular glutathione levels and ferroptosis resistance
ACSL4	Upregulated	Regulates lipid peroxidation and iron metabolism
ALOX15	No difference	Regulates lipid peroxidation
ATL1	No difference	Regulates lipid peroxidation
ATP5MC3	/	Is associated with ferroptosis-related genes
CARS1	No difference	Regulate the expression of ferroptosis-related genes
CS	No difference	Regulate energy metabolism and oxidative stress
DPP4	Upregulated	Regulate the interactions of key genes such as p53 and ACSL4
GLS2	No difference	Regulate energy metabolism and oxidative stress
LPCAT3	No difference	Regulates lipid metabolism
NCOA4	Upregulated	Regulates ferritin degradation and iron release
RPL8	No difference	Interactions with genes related to iron and lipid metabolism.
TFRC	Upregulated	Regulates intracellular iron levels

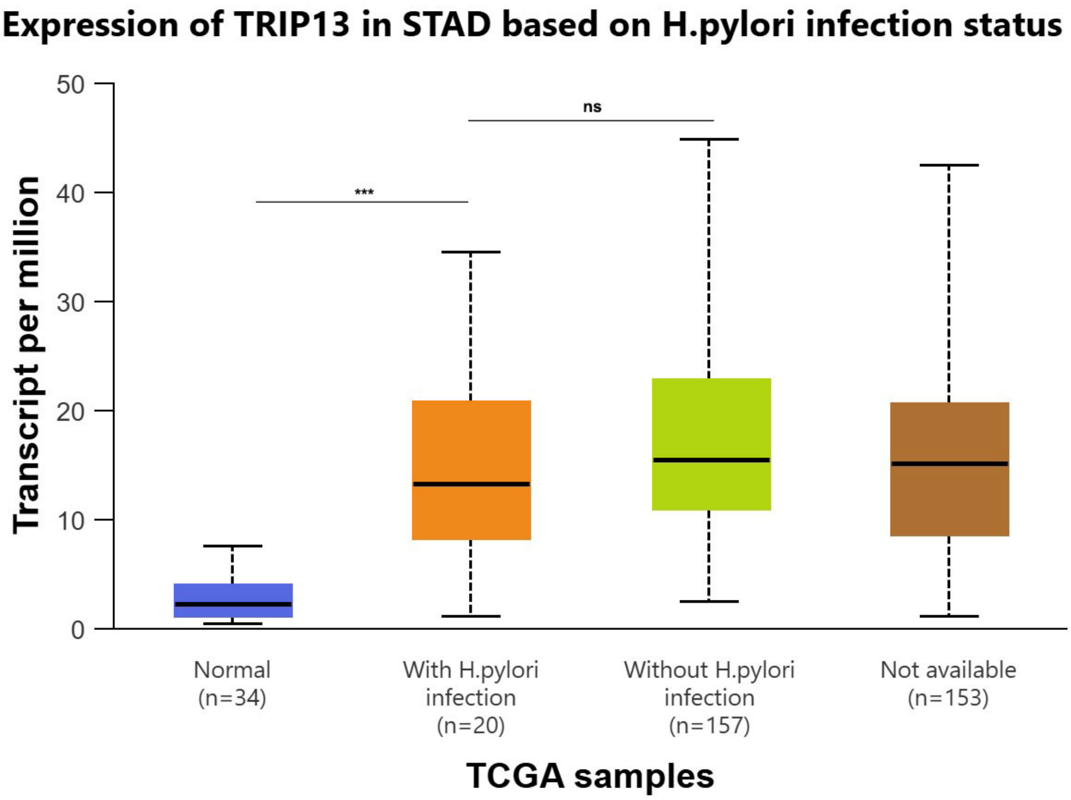

Figure S1. The association between TRIP13 expression and H. pylori-related GC. There is no significant difference in TRIP13 expression levels between gastric cancer patients with and without H. pylori infection. ***$p < 0.001$. ns, no significant change.
